# Epigenetic Alterations That Are the Backbone of Immune Evasion in T-cell Malignancies

**DOI:** 10.7759/cureus.51662

**Published:** 2024-01-04

**Authors:** Mihaela Andreescu

**Affiliations:** 1 Hematology, Colentina Clinical Hospital, Bucharest, ROU

**Keywords:** prognosis, early detection, clinical diagnostic, infectious diseases, molecular biology

## Abstract

Epigenetic alterations are heritable and enduring modifications in gene expression that play a pivotal role in immune evasion. These include alterations to noncoding RNA, DNA methylation, and histone modifications. DNA methylation plays a crucial role in normal cell growth and development but alterations in methylation patterns such as hypermethylation or hypomethylation can enable tumor and viral cells to evade host immune responses. Histone modifications can also inhibit immune responses by promoting the expression of genes involved in suppressing normal immune function. In the case of T-cell lymphoma, adult T-cell lymphomas (ATL) also undergo immune evasion through the exceptional function of its accessory and regulatory genes. Epigenetic therapies are emerging as a promising adjunct to traditional immunotherapy and chemotherapy regimens. Clinical trials are currently investigating the use of epigenetic therapies in combination with immunotherapies and chemotherapies for more effective treatment of ATL and other cancers. This review highlights epigenetic alterations that are widely found in T-cell malignancies.

## Introduction and background

T-cell malignancies are a diverse group of cancers arising from abnormal T-cell development and function. These malignancies include conditions such as T-cell lymphomas, T-cell acute lymphoblastic leukemia (T-ALL), and peripheral T-cell lymphomas (PTCLs) [1]. Despite advances in our understanding of the molecular basis of T-cell malignancies, effective therapeutic strategies remain limited. One critical aspect contributing to the pathogenesis of these diseases is immune evasion which can involve various genetic and epigenetic modifications. Epigenetic alterations have emerged as key players in mediating immune evasion in T-cell malignancies, providing a molecular basis for the immune-resistant phenotype observed in these diseases. Epigenetic alterations are heritable and stable changes in the gene expression that are independent of changes in the underlying DNA sequence [2]. These alterations play a crucial role in determining the characteristics of cells and allow cells with the same genes to develop into different types. Epigenetics is an important phenomenon for controlling when and how genes are turned on or off during processes like development, cell growth, and response to changes in the body or the environment [3]. It is a way for the environment to influence gene expression and impact the risk of certain genetic diseases [4]. Environmental factors including pollution, toxicants, inflammation, and nutrients can encourage epigenetic alterations, which in turn can lead to various disorders, including autoimmune disorders, diabetes, and cancers [3,5]. Epigenetic alterations include noncoding RNAs, histone modifications, and DNA methylation.

Recent studies have shown that epigenetic alterations have an impact on these immune responses [6,7]. The epigenome plays a critical role in the normal development of lymphocytes and the regulation of immune responses [8]. Immune evasion is an approach used by tumors and pathogens to avoid the immune response of the host, promoting their growth and transmission to new hosts [9]. Tumors use various tactics to escape the immune response. They can activate processes that prevent cell death and release substances that suppress the immune system in the area around the tumor. Some of these substances are transforming growth factor (TGF)-β, interleukin (IL)-10, indoleamine 2,3-dioxygenase, and vascular endothelial growth factor (VEGF). These substances contribute to the loss of tumor antigen expression and the potentiation of immune-suppressive lymphocytes such as tumor-associated macrophages, regulatory T-cells, and myeloid-suppressive cells. Additionally, the expression of inhibitory molecules plays a role in immune evasion [10].

T-cell malignancies are highly malignant and aggressive diseases with poor clinical outcomes. Epigenetic modifications play a critical role in their development and pathogenesis by regulating gene expression and signal transduction [11]. Epigenetic alteration leads to immune evasion in several ways, including changes in DNA methylation patterns. DNA methylation involves the addition of a methyl group to the cytosine base of DNA. Alterations in DNA methylation, such as hypomethylation or hypermethylation, can lead to immune evasion. Furthermore, the addition or removal of chemical groups to histone proteins can alter their structure and gene expression, leading to changes in immune responses. Histone deacetylation and altered gene expression also contribute to immune evasion [11]. Non-coding RNAs have also been found to play a key role in the alteration of immune responses. Epigenetic changes, inherited across generations can contribute significantly to the alteration of immune responses. The term used for this purpose is transgenerational inheritance [12]. Understanding the genetic and epigenetic components of immune evasion is crucial. Studies on the modulation of epigenetics and immune checkpoints have shown an interaction between immune modulation and epigenetic alterations [13]. This review article provides a comprehensive summary of the latest research on the epigenetic modifications that can result in immune evasion and the development of T-cell malignancies. The article is of great significance as it highlights the emerging importance of epigenetic mechanisms in T-cell malignancies and their potential role in the development of novel therapeutic approaches.

## Review

Epigenetic alterations

In the modern era, the Human Genome Project is one of the most remarkable accomplishments that sheds light on various surprising mechanisms encoded within the genome. Among these mechanisms, epigenetics has emerged as a crucial factor that represents the chemical interactions and regulatory systems governing genetic code expression [14]. Currently, regulation of tissue-specific expressions, X chromosome inactivation, or genomic imprinting are the main roles of epigenetic modifications. Additionally, the variability of epigenetic alterations in human disorders particularly cancers has further highlighted the significance of epigenetic regulation mechanisms [2]. Recent breakthroughs in understanding these mechanisms have refined the definition of epigenetics from a process of the development of a fertilized zygote into a mature organism to an emphasis on the heritability of traits and gene expression mechanisms [15]. Epigenetics is now understood to control both normal and abnormal events in organisms [16]. One of the main activators of epigenetic alterations is environmental stimuli. Chronic inflammation and aging have been identified as activators of aberrant DNA methylation [17,18]. Cigarette smoking is also known to induce invitro abnormal DNA methylation [19]. In the case of cultured epithelial cells of mammals, estrogen treatment is also a well-known abnormal DNA methylation accelerator [20]. 

Epigenetics of normal cells

The epigenetic landscape of normal cells is established during development and is maintained throughout the lifetime of an organism. It plays a critical role in cellular identity and function by determining which genes are active or inactive in different cell types. In mammals, epigenetics is an essential mechanism for the development of normal cells and the maintenance of gene expression (tissue-specific) patterns [21]. Chromatin is a set of nucleosomes, that consist of 146 base pairs of wrapped DNA around an octant of four core histone proteins. these are H2A, H2B, H3 and H4 [22]. Epigenetics that modifies the structure of chromatin can be classified into four classes. These categories include DNA methylation, covalent histone modifications, non-covalent mechanisms, and non-coding RNAs [23]. Each of these categories exerts unique and interconnected effects on gene expression. DNA methylation is one of the most well-studied epigenetic modifications. It can lead to gene silencing by blocking the binding of transcription factors or recruiting proteins that inhibit gene expression. It plays a crucial role in processes such as genomic imprinting, X-chromosome inactivation, and the maintenance of cellular identity during development [24]. Covalent histone modifications involve the addition or removal of various chemical groups to the tails of histone proteins, which are structural components of chromatin. These modifications include acetylation, methylation, phosphorylation, ubiquitination, and others [25]. Non-covalent mechanisms of chromatin regulation involve ATP-dependent remodeling complexes that can modify the physical structure of chromatin. These complexes use the energy derived from ATP hydrolysis to alter nucleosome positions, remodel chromatin fibers, and create accessible regions for gene regulation [26]. By changing the accessibility of DNA, non-covalent mechanisms play a vital role in controlling gene expression and ensuring proper cellular functioning. Non-coding RNAs (ncRNAs) are RNA molecules that do not code for proteins but have regulatory functions. They can be broadly categorized into two groups: small ncRNAs and long ncRNAs [27]. Understanding the epigenetic modifications that modify the structure of chromatin in normal cells is of great importance. These modifications are not static but dynamically regulated during development.

Epigenetics of abnormal cells

Changes in DNA Methylation

**Figure 1:**
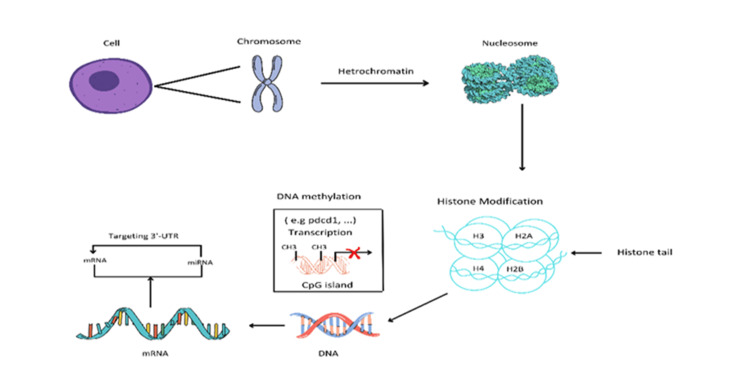
DNA Methylation Backdrop in Normal and Tumor Cells

The epigenetic profile of cancer cells is different from that of normal cells. There are two types of changes in DNA methylation. Hypomethylation (DNA with less amount of DNA methylation than normal cells) and hypermethylation (DNA with more amounts of DNA methylation). In cancer cells, across much of the genome, a decreased DNA methylation occurs [28]. This decreased DNA methylation consequences in the alteration of many of the activities of the genes. This is because methylation is linked to low gene activities but in the case of hypomethylation the activity of genes affected is increased [29]. Genes that control cell differentiation and growth typically display less methylation and higher levels of activity, making them prime candidates for the development of cancer [30]. On the other hand, hypermethylation-associated silencing of tumor suppressor genes is a more limited phenomenon that can occur at specific points or hotspots in the genome. In cancer cells, DNA hypermethylation primarily affects tumor suppressor genes which lead to decreased gene activity and subsequent cancer development [31]. Such hypomethylated and hypermethylated cells tend to develop and grow faster than the normally methylated cells. They grow in abundance and take over the whole population [32]. However, the specific DNA methylation profiles that lead to cancer development can vary greatly between different types of cancer. For instance, while the BRCA1 gene is hypermethylated in ovarian and breast tumors, it is demethylated in other types of tumors [33]. DNA methylation profile in both normal and tumor cells is shown in Figure 1.

Immune Evasion Due to DNA Methylation

DNA methylation leads to the silencing of transcription genes, either by recruiting the chromatin-modifying protein or directly inhibiting the transcription factors binding to suppress the gene expression and chromatin structure [34]. Malignant cells evade detection and attack by natural killer cells and T-cells of the immune system [35]. One of the main immune evasion mechanisms through DNA methylation is the suppression of genes encoding cancer antigens. These antigens are recognized by the immune cells as a foreign agent, after being expressed by the tumor cells [36]. These foreign agents trigger immune responses. However, the silencing or suppression of these antigen-coding genes through DNA methylation renders tumor cells invisible to the immune system, as these genes do not express antigens that can activate the immune system [37].

In a study by Jung et al. [38], they proposed that DNA methylation aberrations play a crucial role in determining how tumors respond to the host immune system. They suggested that such aberrations help highly mutated and rapidly dividing cancer cells to evade the immune system and resist immune therapies. The strategic mechanism involves the heterochromatin formation which is then coupled with an advanced level of methylation loss. This in turn provides support to cancer evolution as they help the cancer cells to evade the immune cells and aid in the fitness of such cells [38]. In another study by Li et al., they reported that the inactivation of histone H3k36, methyltransferase NSD1 induces DNA hypomethylation. This results in reduced immune infiltration of tumor cells. Silencing of genes if innate immunity occurs upon loss of NSD1, these include Type 3 interferon IFNLR1 receptor, through H3K36diemethylation depletion gain of tri methyl H3K27 [39].

Downregulation of Antigen Processing

DNA methylation also leads to the deregulation of genes responsible for the presentation and processing of genes. Macrophages and dendritic cells rely on the genes to present and process cancerous antigens to T-cells [40]. On silencing of these genes, cancer cells become resistant to the immune elimination and recognition system. DNA methylation also causes a suppression of chemokines and cytokines expression, which are a necessity of immune cell recruiting to the site of tumor growth or infection [41]. If these signals are not provided to the immune system, it is unable to locate and recognize the tumor cells in the body. This eventually leads to cancer progression and immune evasion [42]. This powerful mechanism of DNA methylation is used by cancer cells to evade the immune destruction and recognition process. Therefore, it is important to identify and target the immune evasion mediated by DNA methylation, this might help in developing new therapies for cancer and empower the immune system to fight against such cells [43].

Li et al., in 2021, identified that aberrant DNA methylation of PPP2R2B results in tumor suppression of triple-negative breast cancer (TNBC) cells. Analysis was done schematically through bioinformatics. Pieces of evidence obtained through transcriptome, in-vitro experiments, and genome analysis supported that the downregulation of PPP2R2B could assist TNBC cells in immune evasion by suppressing the immune response against tumors. Inclusively, PPP2R2B could be a favorable biomarker in the case of TNBC. It also helps in predicting responses to immunotherapies and direct modified TNBC treatment strategies [39].

Modification in Histone

Histone methyltransferases (HMTs) carry out the methyl group addition to the histones. The histone demethylase (HDM) function is opposite to the HMTs. In case of abnormal cell development, methyl groups are placed at the wrong spot when HMT functions are altered, this leads to the silencing of tumor-suppressing genes [44]. In the same way, HDMS activity is also affected and leads to increased oncogenic activity. Histone acetyl markers are lost in the epigenetics of cancer cells because of increased histone deacetylation [45]. The change of this protein will modify the link between DNA and histones and the shape of complexes of DNA and histones. The methylation effect on the activities of genes varies in according to the amino acid variability. Methyl marks are either regarded as repressing or activating based on their dependence on gene activity. However, there is an interesting turnover that few HDMs can eradicate both repressing and activating marks [46].

Modification of Histones That Cause Immune Evasion

Immune evasion is also affected by histone modification through an alteration of gene expression. Such dysregulated genes include chemokines, antigen-presenting molecules, and encoding cytokines. Changing the DNA accessibility and transcription binding factors to a certain genetic point. The expression of genes is regulated by the histone modifications in two forms: either suppression of immune-responsive genes or their promotion [47]. Steinbach and Riemer have reviewed the human papillomavirus (HPV) immune evasion mechanism. HPV active immune evasion is mediated intracellularly through disturbed functions of proteins and altered gene expression and interfering extracellularly with immune networks from antigen-presenting cells to T cells. Suppressed IFN and cGAS-STING reaction inhibits the antiviral state induction. Downregulation of adhesion molecules and TLRs plus decreased chemokine production by infected keratinocytes pave the path of reduced antigen-presenting cell attraction and thus resulting in a delayed immune response to Anti-HPV. Interference with antigen processing and low protein expressions add a lot to decreased HPV epitope performance. All these mechanisms help in the persistence of HPV for a long time until it completes its life cycle. But this in turn increases lesion persistence risk and malignant transformation onset [48].

*Inhibition of Immune Responses* 

Histone modification can inhibit immune responses by promoting the gene expression involved in inhibiting the immune mechanism [49]. For example, tumor cells can alter histones to overpower the gene expression that encodes the major histocompatibility complex molecules (MHC), which are crucial for presenting the foreign antigen to the T-cells [50]. Histone modification leads to a suppression of MHC encoding gene expression, so tumor cells escape recognition from the immune system, and skip T-cell destructive mechanism. PD-L1 inhibits the activation of T cells and hence prevents the immune response [49]. A blockage of T-cell functionality can help lymphomas evade the recognition and destruction of the immune system [51].

Inhibition of Immune Activation

Histone modifications can also evade immune responses by suppressing the immune-activating gene expressions specifically, in the case of autoimmune disorders. Histone modifications can inhibit the expression of chemokines and cytokines, which are responsible for promoting immune responses [52]. This will result in a reduction of immune responses and tissue damage will also be reduced, which allows the disorder to persist in the body. Moreover, in case of viral infections, viruses alter histone and this alteration will suppress interferon-stimulated expression which is crucial for the antiviral response of the immune system. Thus viruses evade the immune responses, detection, and destruction by the T-cells [49].

Histone deacetylases (HDACs) eliminate the acetyl group from non-histone and histone proteins and play the role of transcriptional repressor. Yeon et al., in 2020, in their study predicted that HDACs are frequently dysregulated in malignancies, affecting the regulation of MAPK signaling, progression of cancer cells, and reaction to several anti-tumor drugs. HDACs have been known to regulate the PD-1/PD-L1 expression and genes that contribute towards immune evasion [53]. In short, immune inhibition, and activation-related gene expression can be regulated by histone modifications. Considering the biological relevance of histone modifications for immune evasion, this may lead to the development of new autoimmune and cancer therapies, possibly affecting host and virus interactions [54].

MicroRNAs (mRNAs) and Immune Evasion

miRNAs are short, endogenous 19-25 nucleotide long, non-coding RNAs that perfectly or partially match the target messenger RNA 3′ untranslated regions (3′UTR) for the regulation of expression of genes through post-transcriptional silencing and degradation of targeted mRNAs [55]. Experimental and bioinformatic studies have shown that miRNAs have a role in all the processes of life such as cell growth, apoptosis, regulation of cell cycle, stress reaction, and cell differentiation as 30% of the human genes are directly targeted by miRNAs. Sanger miRNA's latest registry annotates that there are more than 800 miRNAs in humans and several more miRNAs will surely be identified in the future [56].

In normal cells, just like for other protein-coding genes, miRNAs contribute to the normal cell transcriptome and undergo tight regulation while in the case of lymphomas, they are found to be highly deregulated and massively expressed. The interaction of epigenetic mechanisms and miRNA is a complex regulatory system [57]. There are also pieces of evidence suggesting that miRNAs are tissue-specific and can affect epigenetic mechanisms such as histone modifications and DNA methylation, and that regulate gene transcription and post-transcriptional gene silencing [57,58]. 

T-cell Malignancies and Epigenetic Alterations

T-cell lymphoma is a malignancy of T-cells and mature CD+4 cells. This is mainly caused by the T-cell leukemia virus Type 1 (HTLV-1) [59,60]. In comparison with the human immune deficiency virus (HIV) (pathogenic retrovirus), HTLV-1 replication level is low in vivo, and virions from HTLV-1 transmission are not very efficient [61]. Suppression of infected cell death and clonal proliferation occurs as a result of HTLV-1 persistent infection. Immune evasion is also achieved by this virus through the exceptional function of its accessory and regulatory genes. The survival and proliferation of infected cells lead to the accumulation of epigenetic and genetic aberrations in the genes of the host cells [61].

Immune Evasion of T-Lymphoma

Host immunity prevents the development of adult T-cell lymphoma (ATL), due to the immune response towards the HLTV-1. Host immunity responses are seen in approximately 90% of the ATL cases [60]. Strong T-cell responses are recorded in patients who receive a very stressful treatment such as hematopoietic cells [62,63]. Reported results have indicated the presence of an anti-tumor or anti-viral immune response against the development of T- T-cell lymphomas [60]. Despite the immune responses to this viral disease, it has been reported in a recent study that cells of ATL can escape from natural killer (NK) cells mediated immunity through the deregulation of CD48 [64]. This observation suggests that gene silencing for viral expression in cells of ATL and defects in the anti-viral immune responses permits the HTLV-1 infected cells to induce immune evasion, survive, and eventually transform into clones [60].

Epigenetic Regulators of T Lymphocytes

Epigenetic aberrations can lead to transcriptional dysregulations in all types of lymphomas. Epigenetic changes result in silencing of tumor suppressor genes through their promoter regions. This is an important mechanism in the case of oncogenesis and several other such genes [65]. These include miR-31, p16 INK4A, NDRG2, and TCF-8, these genes are well known for their deregulation in ALT cells due to repressive histone modifications or CpG hypermethylation [66]. In this way, ALT cells evade the host's immune response and survive [60]. Other main epigenetic regulators of lymphomas include EZH2, KMT2, CREBBP, ARID1A, DNMTA, TET2, and IDH2 [67].

Immunotherapy Challenges

Identification of biomarkers that predict clinical responses to PD-1/PDL1 and CTLA-4 blockade is one of the major challenges faced by current immunotherapies [38]. Somatic copy number alterations (SCNAs), genetic alterations of certain types of genes, or in pathways, and tumor heterogeneity have been recognized as the resistance factors of immunotherapies [68,69]. Global methylation also counteracts with checkpoint blockade clinical advantages. On the other hand, neoantigen or mutational load and existing T-cell infiltrations are thought to be positive predicting factors for the clinical advantage of blockade checkpoints [70]. Chen et al. have hypothesized that treatment with a hypomethylating (HMA) agent would help in the induction of an antitumor immune response to sensitize people suffering from ovarian cancer to the anti-PD-1, immunotherapy. Phase 2 clinical trial was performed by the authors to test the combination of a second-generation HMA, guadecitabine, with an immune PD-1 checkpoint inhibitor, pembrolizumab.

The clinical trial was performed on 35 platinum-resistant patients with ovarian cancer. The desired result was not attained from the immune checkpoint blockade and HMA, but correlation analysis gave information about which immune therapy will be beneficial for people with ovarian cancer [71]. Cellular adoptive therapy, dendritic cell vaccines, and some other strategies have yet to display success for a broad number of tumor cell types. As we stated earlier cancer cells become resistant to immune therapy by intrinsic and extrinsic factors in tumor cells that lead to immune evasion. Extrinsic factors include immune suppressive cells such as T regulatory cells, myeloid-derived suppressor cells, and tumor-associated macrophages. These cells produce immune suppressive effects by secreting inhibitory ligands for interaction with receptors of T-cells such as CTLA-4 and PD-1. In immune therapy, both acquired and primary resistance are a problem. However, PD-1 and CTLA-4 immune checkpoint blockade therapies have shown some success in immune activation enhancement [35].

Prospects of epigenetic treatment of lymphomas

From all the above discussion, it is clear that several studies have shown that epigenetic aberrations are a leading cause of the development and spread of lymphomas. Achievement of a therapeutic effect in the case of lymphomas involves cellular reprogramming [72]. Epigenetic monotherapy has shown promising results in recent clinical trials. This has led many epigenetic agents to be approved for use in the treatment of lymphoma. Nowadays, immunotherapies and chemotherapies are being used in combination with epigenetic therapies in several clinical trials [73].

This combination therapy is being extensively studied preclinically and is being used to overcome the limitations of current monotherapies due to cell signaling inhibition by redundant pathways. Examples of combinations being currently tested include BET inhibitors individually used in combination with small molecule inhibitors against HDACs, EZH2, ATR, BTK, P13K, mTOR plus lenalidomide [74,75]. Similarly, decitabine has been combined with BET143, AKT, JAK-STAT, and BCL2 inhibitors. A substantial number of such combinations are being studied at the preclinical level and some of them have reached the animal preclinical testing level, before their application in humans. The most thrilling avenue for future research studies is the combination of immunotherapy with epigenetic modulating agents [73].

## Conclusions

Epigenetic alterations are the backbone of several immune evasion mechanisms. Alteration in epigenetics leads to several autoimmune disorders, diabetes, and lymphomas. Normal epigenetic mechanisms include DNA methylation, covalent histone modifications, non-covalent mechanisms, and non-coding RNAs. Immune evasion results from alteration in DNA hypomethylation and hypermethylation of both leading, via different mechanisms, to suppressing the expression of those genes responsible for detecting foreign cells and inducing an immune response. Antigen downregulation is also seen in the altered DNA methylation process. Similarly, epigenetic changes lead to either the suppression or activation of chemokines and cytokines expressions, resulting in the survival and development of tumor cells. T-cell lymphomas are also capable of immune evasion due to antiviral gene suppression in the immune system. Epigenetic therapies are also emerging in addition to the traditional immunotherapy and chemotherapy regimens as a striking add-on. These merging therapies work in synergy with other treatments and offer low toxicity. Further research is required on the novel combinations of inhibiting agents, and therapeutic roles, and to identify new epigenetic pathways.
